# The ER mitochondria calcium cycle and ER stress response as therapeutic targets in amyotrophic lateral sclerosis

**DOI:** 10.3389/fncel.2014.00147

**Published:** 2014-05-30

**Authors:** Vedrana Tadic, Tino Prell, Janin Lautenschlaeger, Julian Grosskreutz

**Affiliations:** Hans Berger Department of Neurology, Jena University HospitalJena, Germany

**Keywords:** amyotrophic lateral sclerosis, ER stress, protein misfolding, calcium dysregulation, SOD1, TDP-43, mitochondria, oxidative stress

## Abstract

Amyotrophic lateral sclerosis (ALS) is a neurodegenerative disease characterized by progressive loss of upper and lower motor neurons. Although the etiology remains unclear, disturbances in calcium homoeostasis and protein folding are essential features of neurodegeneration in this disorder. Here, we review recent research findings on the interaction between endoplasmic reticulum (ER) and mitochondria, and its effect on calcium signaling and oxidative stress. We further provide insights into studies, providing evidence that structures of the ER mitochondria calcium cycle serve as a promising targets for therapeutic approaches for treatment of ALS.

## INTRODUCTION

Amyotrophic lateral sclerosis (ALS) is a fatal neurodegenerative disorder characterized by progressive degeneration of the upper (spasticity, dysphagia, dysarthria) and lower motor neurons (atrophy, fasciculations). Approximately 90% of ALS patients have sporadic ALS (sALS) which is the most prevalent form and about 10% have the inherited or familial form of ALS (fALS). The latter form is believed to be due to several genes including *SOD1*, *TARDBP, FUS, OPTN, *and *VCP*. In addition, a hexanucleotide (GGGGCC) repeat expansion in the first intron of the *C9ORF72* gene (DeJesus-Hernandez et al., 2011; Renton et al., 2011) has lately been demonstrated as being associated with ALS. However, the etiology of the disease is still unclear, although recent studies indicate that calcium (Ca^2+^) disturbances, ER stress, and mitochondrial dysfunction are involved in the pathogenesis of ALS (Grosskreutz et al., 2010; Lautenschlaeger et al., 2012). Other mechanisms possibly involved in ALS-related pathophysiology comprise: oxidative stress, protein aggregation, dysregulated endosomal trafficking, impaired axonal transport, neuroinflammation, and dysregulated transcription and RNA processing (Ferraiuolo et al., 2011). Several properties of motor neurons make them more vulnerable than other neuronal groups. Motor neurons express high levels of Ca^2+^–permeable α-amino-5-methyl-3-hydroxisoxazolone-4-propionate (AMPA) receptors that lack the GluR2 subunit which makes them more vulnerable to excitotoxicity and dysregulation of intracellular Ca^2+^ homeostasis (Williams et al., 1997). Also, low levels of Ca^2+^ -buffering proteins contributes greatly to this vulnerability (Ince et al., 1993). Because of high metabolic demands, motor neurons are largely dependent on optimal mitochondrial function, a robust cytoskeleton and an axonal transport mechanism. Despite all the above facts, there remain numerous unanswered questions in ALS related to selectivity and specificity of the cellular targets of motor neuron degeneration and cell-specific aspects of mitochondrial Ca^2+^ signaling. This review focuses on crosstalk between ER, mitochondria, oxidative stress and calcium.

### ALS GENES AND ENCODED PROTEINS – ROLE IN PATHOPHYISOLOGY

#### Cu/Zn superoxide dismutase 1 (SOD 1)

About 20% of fALS patients carry a mutation in the *SOD1* gene. Indeed, more than 170 different SOD1 mutations have been described in ALS families (http://alsod.iop.kcl.ac.uk/). Generally, *SOD1* mutations have not been linked to decreased SOD activity (Kostrominova, 2010; Fischer et al., 2011), instead mutant SOD1 likely acts through a combination of several mechanisms, including protein misfolding, mitochondrial dysfunction, oxidative damage, cytoskeletal abnormalities, and defective axonal transport, excitotoxicity, in addition to inadequate growth factor signaling and inflammation (Cozzolino et al., 2008).

#### Fused in sarcoma (FUS) and TAR DNA-binding protein (TDP-43)

Mutations in the gene encoding fused in sarcoma/translocated in liporsarcoma (FUS/TLS or FUS) are linked to 4% of fALS cases. Mutations in *TARDBP* (which encodes TDP-43) account for 4% of fALS and a smaller percentage of sALS. The toxicity of TDP-43 and FUS/TSL proteins is linked to their altered intracellular localization. Both TDP-43 and FUS/TLS are mainly localized in cell nuclei where they control gene transcription and pre-mRNA (Buratti et al., 2001; Winton et al., 2008; Kwiatkowski et al., 2009). In the presence of mutations or stress, these proteins accumulate in the cytosol (Liu-Yesucevitz et al., 2010; Dewey et al., 2012; Daigle et al., 2013; Walker et al., 2013; Watanabe et al., 2013). FUS triggers ER stress and causes fragmentation of the Golgi apparatus in patients with fALS (Farg et al., 2013). In NSC34 transfected cells, wild type human TDP-43 caused ER stress (Suzuki et al., 2011). In the same cell line, mutant TDP-43 induced mitochondrial dysfunction and probably caused oxidative stress (Duan et al., 2010). Experiments with TDP-43 mutation in zebrafish resulted in impairment of neuromuscular junctions (Armstrong and Drapeau, 2013).

#### Chromosome 9 open reading frame 72 (*C9ORF72*)

Large expansions of a non-coding GGGGCC-repeat in the first intron of the *C9ORF72* gene are accountable for 40% of fALS. C9ORF72 hexanucleotide repeats form highly stable RNA G-quadruplexes, which probably influence RNA transcription, splicing, translation and transport (Fratta et al., 2012). C9ORF72 pathology is characterized by intracellular inclusions, however the major proteins forming these inclusions have not yet been elucidated (Mori et al., 2013).

#### Other genes

Mutations in the valosin–containing protein (*VCP*) are responsible for 1–2% of fALS cases. In mice, overexpression of mutant VCP produces ubiquitin- and TDP-43-positive inclusions, suggesting that TDP-43 plays a role in VCP-induced disease (Rodriguez-Ortiz et al., 2013). Mutant VCP also impact mitochondria, such as via a decrease in ATP production related to mitochondrial uncoupling (Bartolome et al., 2013).

Another mutated gene found in patients with ALS comprises *OPTN* that encodes the protein optineurin which regulates membrane trafficking, protein secretion, cell division and host defense against pathogens (Kachaner et al., 2012). Wild-type optineurin suppresses nuclear factor-kappa B (NF-κB) activity, but the ALS-causing mutant optineurin is unable to suppress NF-κB activity. Therefore, there is an indication that inappropriate NF-κB activation is the pathogenic mechanism underlying optineurin mutation-related ALS (Akizuki et al., 2013). In two patients carrying mutation in *OPTN*, was shown that loss of function rather than proteinopathy itself resulted in the formation of TDP-43 inclusions in neuronal and glial cytoplasm, and Golgi apparatus fragmentation (Kamada et al., 2014).

Vesicle-associated membrane protein (VAMP)-associated protein B (VAPB) is usually ER-resident and is integral to its structure, protein transport, lipid metabolism, and the UPR. VAPB toxicity is probably mediated by impaired Ca^2+^ homeostasis and ER stress (Langou et al., 2010; De Vos et al., 2012; Morotz et al., 2012).

## THE ERMCC AND CALCIUM DISTURBANCES IN ALS

**FIGURE 1:**
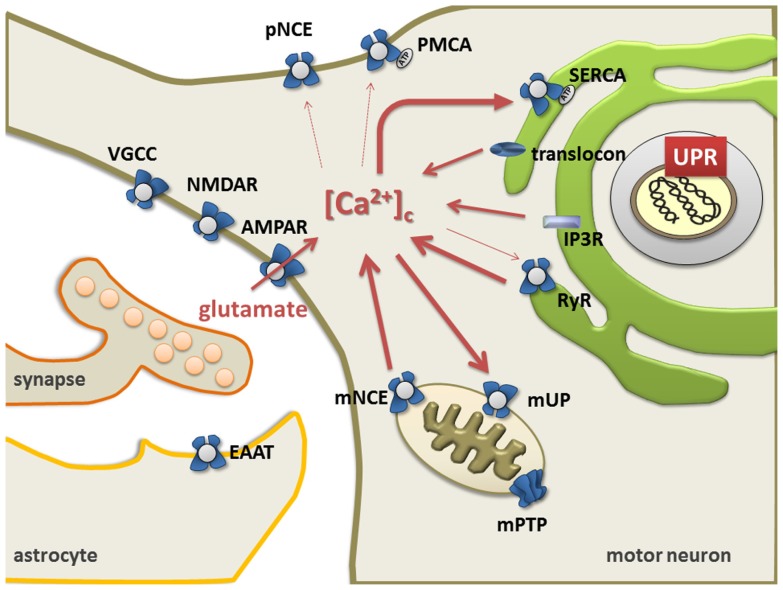
**The endoplasmic reticulum mitochondria Ca^2+^cycle (ERMCC).** Ca^2+^can enter cytosol through: the AMPA receptor, the ryanodine receptor (RyR) at the ER membranes, the opening of the inositol 1,4,5 trisphosphate receptor (IP_3_R), the translocon at the ER membrane, and/or the plasmalemmal voltage gated Ca^2+^channels (VGCC). Triggered by physiological activity of AMPA receptors with pathologically increased Ca^2+^-permeability in ALS, a chronic shift of Ca^2+^ from the ER to the mitochondria (i.e., through Ca^2+^-induced Ca^2+^release through RyR and mitochondrial uptake through the uniporter mUP) causes depletion of ER Ca^2+^levels with protein misfolding (UPR) and chronic mitochondrial Ca^2+^overload. Cytosolic Ca^2+^clearance is facilitated by the plasma membrane Ca^2+^ATPase, the plasmalemmal Na^+^/Ca^2+^exchanger (NCE), the sarco/endoplasmicreticulum Ca^2+^ATPase (SERCA), and the Golgi apparatus. Astrocytes control the level of persisting glutamate at the glutamatergic synapse through glutamate transporters (EAAT), but also exert life-supporting functions in motor neurons (i.e., BDNF, IGF, VEGF). (NMDAR = NMDA receptors, VGCC = voltage gated Ca^2+^channels, Na/K = Na^+^/K^+^pump, pNCE = plasmalemmal Na^+^/Ca^2+^ exchanger, PMCA = plasmalemmal Ca^2+^ATPase, mNCE = mitochondrial Na^+^/Ca^2+^ exchanger, SERCA = sarco-endoplasmic Ca^2+^ATPase). Modified picture taken from (Prell et al., 2013).

The ER and mitochondria form a highly dynamic interconnected network that is involved in the generation of Ca^2+^ signals. Ca^2+^ release from ER is controlled by ryanodine receptors (RyRs, Ca^2+^-gated Ca^2+^ channels) (Meissner, 2002; Lanner et al., 2010), the inositol 1,4,5-triphosphate receptor-gated channels (IP_3_Rs), and the translocon (Taylor and Tovey, 2010). Restocking of the ER with Ca^2+^ is executed by the sarco/endoplasmic reticulum Ca^2+^ ATPase (SERCA; Wuytack et al., 2002; Verkhratsky, 2005; Lipskaia et al., 2009). Ultimately, the plasma membrane Na^+^/Ca^2+^ exchanger and Ca^2+^ ATPase remove Ca^2+^ from the cell (Rhodes and Sanderson, 2009; **Figure 1**).

Mitochondria take up Ca^2+^ via a Ca^2+^-sensitive electrogenic carrier, the mitochondrial uniporter (mUP) which is gated by cytosolic Ca^2+^ in a biphasic-dependent manner (Gunter and Sheu, 2009). Ca^2+^ uptake into mitochondria is facilitated by Ca^2+^/calmodulin. However, sustained cytosolic Ca^2+^ levels inactivate the uniporter, preventing further Ca^2+^ uptake (Moreau et al., 2006). Accumulated Ca^2+^ in the mitochondria can slowly be ejected back into the cytosol through Na^+^/Ca^2+^ and 2H^+^/Ca^2+^ exchangers (Pivovarova and Andrews, 2010; **Figure 1**). Once intramitochondrial Ca^2+^ rises above a certain threshold, the voltage- and Ca^2+^-dependent high-conductance channel in the inner membrane, known as the mitochondrial permeability transition pore (mPTP), opens, leading to cell death either by apoptosis or necrosis (Leung and Halestrap, 2008; Martin, 2010b). Mitochondria contain similar low Ca^2+^ levels as resting cells, but accumulate a considerable amount during stimulated Ca^2+^ entry, which affects numerous cellular processes such as cellular energy metabolism, synaptic transmission and excitability, intracellular signaling, generation of ROS, and activation of apoptosis (Chinopoulos and Adam-Vizi, 2010; Starkov, 2010).

Several studies have previously investigated abnormalities of Ca^2+^ homeostasis, ER and mitochondria as well as excitotoxicity in motor neurons in ALS (Grosskreutz et al., 2010; Lautenschlager et al., 2013). Based on the models described by Berridge (2002), a persistent shift of Ca^2+^ from the ER to mitochondria (i.e., through Ca^2+^induced Ca^2+^ release via RyR and mitochondrial uptake through mUP) was postulated. This could be triggered by the physiological activity of AMPA receptors together with a pathologically increased Ca^2+^-permeability (Grosskreutz et al., 2010). This in turn, leads to a depletion of Ca^2+^ levels in the ER, resulting in protein folding dysfunction and chronic mitochondrial Ca^2+^ overload. Both protein misfolding and Ca^2+^ overload can then induce apoptosis through Bcl-2 dependent mechanisms (Grosskreutz et al., 2010). Since Ca^2+^ appears to be shuttled back and forth between the ER and the mitochondrial compartment, the process has been termed the ER–mitochondria Ca^2+^ cycle (ERMCC, **Figure 1**; Grosskreutz et al., 2010).

## IMPACT OF ER STRESS ON MITOCHONDRIA

**FIGURE 2:**
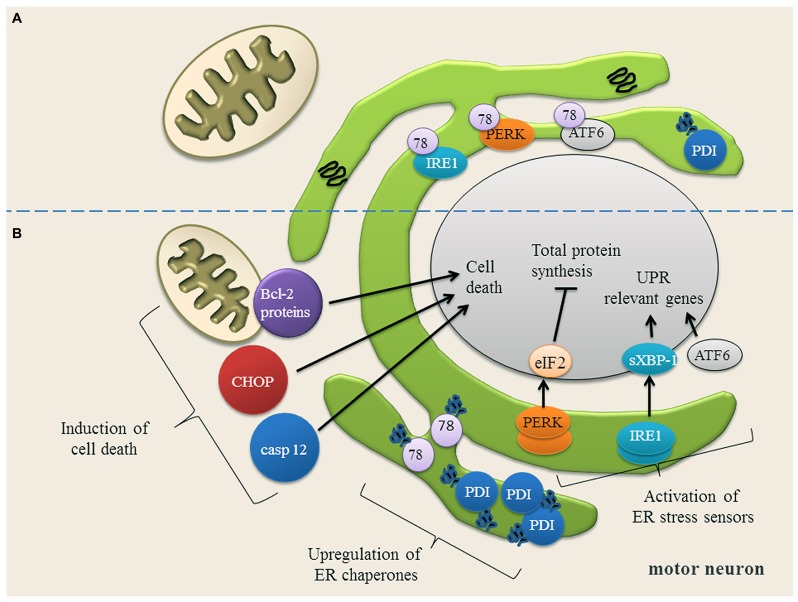
**(A)** Motor neuron in a physiological state and under ER stress conditions. In motor neurons, most proteins in the ER are physiologically properly folded. The ER chaperones Grp78 (78) and protein disulfide isomerase (PDI) are moderately expressed and assist protein folding. The unfolded protein response (UPR) sensor proteins (IRE1, PERK, ATF6) are bound to Grp78 and are therefore in an inactive state. **(B)** Upon ER stress, Grp78 dissociates from IRE1, PERK and ATF6 resulting in the activation and up-regulation of these three UPR sensor proteins. IRE1 splices XBP-1 mRNA (sXBP-1) which up-regulates the expression of UPR relevant genes. ATF6 is transported to Golgi apparatus, where it is cleaved. The cytosolic domain of ATF6 translocates to the nucleus to activate gene expression. PERK phosphorylates eIF2α, thereby down-regulating the total protein synthesis to prevent an overload of the ER. Once the expression of ER chaperones Grp78 and PDI is induced, they bind to the misfolded proteins and try to assist their folding. If restoring of ER homeostasis fails, cell death proteins transcription factor C/EBP homologous protein (CHOP) and caspase 12 (casp 12) are induced and the balance of Bcl-2 family members is disturbed, anti-apoptotic Bcl-2 family members are down-regulated, while pro-apoptotic Bcl-2 family members are up-regulated. Picture modified from (Lautenschlaeger et al., 2012).

Recent studies indicate that ER stress is involved in the pathogenesis of familial and sporadic ALS (Ilieva et al., 2007; Atkin et al., 2008; Walker, 2010; Lautenschlaeger et al., 2012; Prell et al., 2012). ER stress occurs when ER Ca^2+^ content is depleted (Verkhratsky, 2005) and misfolded proteins accumulate in the ER. To cope with ER stress, cells activate the unfolded protein response (UPR; **Figure 2**). The UPR mediates the (1) upregulation of genes encoding ER-resident chaperones, (2) down-regulation of general protein synthesis in order to reduce the ER protein load, and, (3) degradation of misfolded proteins by the proteasome (Kozutsumi et al., 1988; Yoshida et al., 2001; Dudek et al., 2009; **Figure 2**). On a cellular level, ER stress is transduced by three proximal sensors of the UPR: the double-stranded RNA-activated protein kinase (PKR)-like ER kinase (PERK), the basic leucine-zipper transcription factor 6 (ATF6) and the inositol requiring enzyme 1 (IRE1) (Prell et al., 2013; **Figure 2**). When protein misfolding can no longer be compensated for, the prolonged UPR triggers apoptosis by the caspase pathways (Nakagawa et al., 2000; **Figure 2**).

ER stress can affect mitochondria because both organelles are functionally and morphologically connected by several pathways (Vannuvel et al., 2013). In particular, the contact between ER and mitochondria is essential for coordination of the Ca^2+^ transfer (Rowland and Voeltz, 2012). The proteins B-cell lymphoma 2 (Bcl-2), Bcl-2-associated X protein (Bax/Bak) and the Bcl-2-interacting killer (BIK) can enhance Ca^2+^ transfer from ER to mitochondria and the ensuing Ca^2+^ accumulation activates apoptosis via cytochrome C (Germain et al., 2002; Nutt et al., 2002; Fannjiang et al., 2004; Kong et al., 2005). The PERK/ATF4 pathway can induce Lon protease that controls the assembly and/or the degradation of cytochrome C (Margineantu et al., 2002; Venkatesh et al., 2012). Bid, which is a pro-apoptotic BH3-only protein can be cleaved upon ER stress, which subsequently activates caspase-2 or caspase-8 leading to apoptosis (Upton et al., 2008; Uchibayashi et al., 2011). Recruitment of the dynamin-related protein 1 mediates the fission of the outer mitochondrial membrane (Breckenridge et al., 2003).

### THERAPEUTIC STRATEGIES FOCUSED ON ER STRESS

Several therapeutic strategies aim to target the ER (**Table 1**; **Figure 4**). For instance, Salubrinal is a substance that reduces ER stress by activating the UPR. UPR activation is mediated via phosphorylation of elF2α and activation of PERK (Boyce et al., 2005). Salubrinal prevented neuronal cell death triggered by several ER stress inducers (Smith et al., 2005; Reijonen et al., 2008). Moreover, Salubrinal, Guanabenz and Phenazine have all been shown to reduce ER stress in worms and zebrafish expressing mutated TDP-43 (Vaccaro et al., 2013). In SOD1G93A mice, Salubrinal decreased muscle strength loss and extended survival (Saxena et al., 2009).

**Table 1:** Substances targeting ER.

	Proposed mechanism	ALS model	Patients	Experimental evidence	Reference
**ER targets**					
Salubrinal	Reduces ER response	Neuro2a cells transfected with SOD1G93A and SOD1G85R		Salubrinal inhibited dephosphorylation of eIF2α and protected cell from the mutant SOD1-induced death by suppressing UPR	Oh et al. (2008)
		SOD1G93A mice		Salubrinal decreased muscle strength loss and extended survival in SOD1G93A-fast mice	Saxena et al. (2009)
		*C. elegans* and *D. rerio* expressing mutant TDP-43		Reduced paralysis, neurodegeneration, and oxidative stress	Vaccaro et al. (2013)					

Arimoclomol	Co-inducer of the heat shock response under condition of cellular stress	SOD1G93A mice		Arimoclomol delayed muscle denervation followed by rise in expression of heat shock protein 70	Kalmar et al. (2012)
		SOD1G93A mice		Late stage treatment with arimoclomol delayed disease progression and prevented protein aggregation	Kalmar et al. (2008)
			ALS patients	Arimoclomol crosses blood brain barrier and dosage up to 300~mg/day are well tolerated and safe in ALS	Cudkowicz et al. (2008)
			ALS patients	Phase II/III clinical trial for ALS patients with SOD1 mutation	(http://www.clinicaltrials.gov/ct2/show/NCT00706147?term=arimoclomol&rank=1)

PRE-084	Sigma-1 receptor agonist	SOD1G93A mice		Prevented neurons loss possible by activation of protein kinase C and reducing microglia activation	Mancuso et al. (2012)
		Wobbler mice		Increased the levels of BDNF in the gray matter, improved motor neuron survival and ameliorated paw abnormality and grip strength performance	Peviani et al. (2014)

Cyclopiazonic acid	Inhibitor of SERCA	Cultured neurons from SOD1G93A mice		Protective effects against kainate induced excitotoxicity	Lautenschlager et al. (2013)

Another approach to target the ER is to encourage the natural cellular protein-folding machinery via activation of the heat shock transcription factor 1 (Hsf1). Hsf1 is the master activator of chaperone protein gene expression (Neef et al., 2011). Overexpression of human molecular chaperone hHSJ1a *in vivo* mediated late-stage neuroprotection in the SOD1G93A mouse model, probably through a combination of chaperone, co-chaperone and pro-ubiquitylation activity on SOD1 (Novoselov et al., 2013). Arimoclomol, a hydroxylamine derivate and a co-inducer of the heat shock response delayed muscle denervation in the SOD1G93A mouse followed by a rise in expression of the heat shock protein 70 (Kalmar et al., 2012). The therapeutic potential of this drug is under investigation in a phase II/III clinical trial for ALS patients with SOD1 mutations (http://www.clinicaltrials.gov/ct2/show/NCT00706147?term = arimoclomol&rank=1). Other available pharmaceuticals that up-regulate heat shock response and that may be used to treat ALS include Celastrol and 17-AAG (Kalmar et al., 2014).

Accumulation of misfolded proteins may also be targeted by small molecule regulators of autophagy such as antipsychotics (fluspirilene, trifluoperazine, pimozide) and calcium-channel modulators (nicardipine, niguldipine, amiodarone; Sarkar et al., 2007; Zhang et al., 2007).

The Sigma-1 receptors have also gained attention in the recent past. The receptors are a chaperone proteins residing at the mitochondrion-associated ER membrane, where they affect mitochondrial Ca^2+^ influx by stabilizing IP_3_R and acting as inter-organelle signaling modulators of Ca^2+^ homeostatasis, ER stress and apoptosis. The Sigma-1 receptor agonist PRE-084 prevented neurons loss in SOD1G93A transgenic mice, probably by the activation of protein kinase C and reducing microglia activation (Mancuso et al., 2012). Neuroprotective effects of PRE-084 have also been demonstrated in the wobbler mouse model not linked to SOD1 mutation that is characterized by progressive neural atrophy shortly after birth (Peviani et al., 2014). Another agonist of Sigma-1 receptor SA4503 prevented SOD1G93A–induced neurotoxicity in NSC34 cells and extended survival of SOD1G93A mice (Ono et al., 2014). Pharmacological manipulation of the Sigma-1 receptor may increase availability of growth factors, as well as the modulation of astrocytosis and of macrophage/microglia as part of the mechanism involved in Sigma 1 receptor-mediated neuroprotection (Peviani et al., 2014).

Treatment with Geldanamycin, an inducer of heat shock response, successfully blocked protein aggregation but not Ca^2+^ dysregulation or loss of mitochondrial membrane potential (ΔΨ) in murine motor neurons expressing human SOD1G93A (Tradewell et al., 2011). This implies chaperone-based therapies would possibly require co-therapy targeting other important mechanisms of toxicity.

## CROSS-TALK BETWEEN CALCIUM, MITOCHONDRIA, AND REACTIVE OXYGEN SPECIES SIGNALING

### MITOCHONDRIAL DYSFUNCTION

**FIGURE 3:**
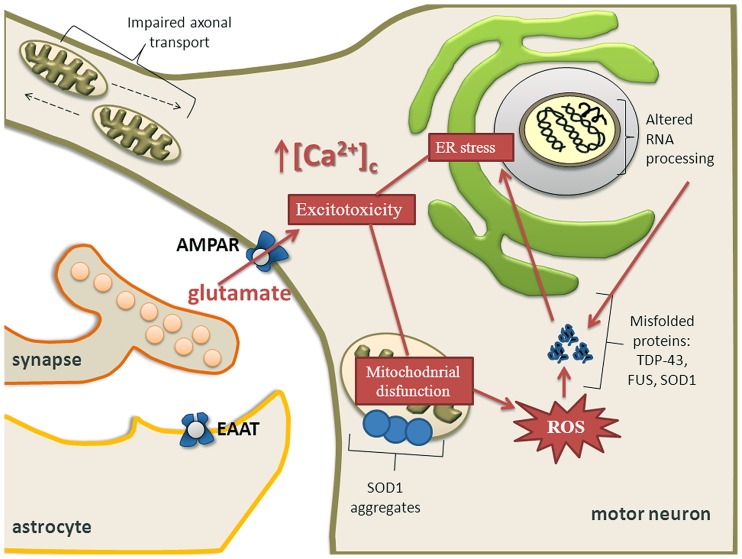
**Calcium dysregulation, ER stress and mitochondrial impairment are major components of excitotoxicity in motor neurons.** Mitochondrial dysfunction causes activation of proteolytic and ROS generating-generating enzyme systems. Mutant SOD1 forms insoluble aggregates in mitochondria at the surface of the outer membrane. Motor neuron might also undergo transcriptional dysregulation and abnormal RNA processing which together with depleted ER Ca^2+^ levels and overproduction of ROS contribute to aberrant protein folding. Aberrant proteins form aggregates leading to ER stress and ultimately activating apoptotic pathways, especially when the unfolded protein response is exhausted. Impaired axonal transport may also contribute to an energy deficit in the distal axon and the dying back axonopathy that is observed in ALS.

Mitochondria are central for energy metabolism and have been well studied in relation to ALS pathogenesis (von Lewinski and Keller, 2005; Cozzolino and Carri, 2012; Jaiswal, 2013). The levels of mutated mitochondrial DNA (mtDNA) were higher in ALS patients but the amount of mtDNA was reduced compared to controls. This reduction correlated well with a decrease of a mitochondrial marker, citrate synthase activity and with the activities of respiratory chain complexes I + III, II + III, and IV, suggesting a loss of mitochondria in ALS spinal cords (Wiedemann et al., 2002). Activity of cytochrome C oxidase in mitochondria is reduced in the spinal cord of sALS patients (Borthwick et al., 1999) and ALS spinal neurons show varied and reduced mtDNA gene copy numbers and increased mtDNA gene deletions (Keeney and Bennett, 2010). Oxidative stress, protein nitration and aggregation, and excitotoxicity participate in the process of motor neuron degeneration caused by mutated SOD1 (Martin et al., 2007). One of the pathological hallmarks of ALS is aggregation of ubiquitinated proteins in motor neurons (Stieber et al., 2000; **Figure 3**). SOD1, FUS, TDP-43, OPTN, and UBQLN2 have been identified as forming aggregates. Whether there is a causal relationship between misfolded proteins and mitochondrial dysfunction for novel mutations is still largely unknown, but there is considerable body of literature describing SOD1 and more recently TDP-43. Mutant SOD1 forms insoluble aggregates in mitochondria at the surface of the outer membrane (**Figure 3**). Further, there is direct connection between mutated SOD1 and impaired mitochondrial function (Liu et al., 2004; Pasinelli et al., 2004; Pickles et al., 2013). Bcl-2 has been identified as an interacting partner of mutated SOD1 because SOD1 induces mitochondrial morphological changes and impairs mitochondrial membrane integrity only in the presence of Bcl-2. This leads to the release of cytochrome C, ultimately leading to cell death (Pedrini et al., 2010).

Degenerating mitochondrial vacuoles have been reported in presymptomatic mice expressing mutant SOD1 in previous studies (Wong et al., 1995; Kong and Xu, 1998). However, mitochondrial disturbances are not restricted to SOD1 mutations. In patients with ALS, dense conglomerates of mitochondria have been found in the anterior horn of lumbar and spinal cord and proximal axons (Hirano et al., 1984; Sasaki and Iwata, 1996). It has been demonstrated that neuronal Ca^2+^, mitochondrial volume and a number of synaptic vesicles are increased in ALS patients (Siklos et al., 1996). In addition, overexpression of TDP-43 causes mitochondrial dysfunction and induces mitophagy (Hong et al., 2012) and oxidative injury in NSC34 cell line (Duan et al., 2010; Lu et al., 2012). In a yeast model, TDP-43 aggregates around mitochondria and there is an inverse correlation between respiratory activity and toxicity of the mutant protein (Braun et al., 2011). Overexpression of wild-type TDP-43 resulted in reduced mitochondrial length and density in neurites of primary motor neurons and conversely, suppression of TDP-43 resulted in significantly increased mitochondrial length and density in neurites (Wang et al., 2013). Neuronal mitochondrial transport and morphological abnormalities occur *in vivo* in SOD1 and TDP-43 ALS (**Figure 3**) mouse models but show differences in temporal and spatial manifestation. This implies that different molecular mechanisms may be involved (Magrane et al., 2013).

### MITOCHONDRIAL CALCIUM DYNAMICS

#### Ca^2+^ regulation of mitochondrial metabolism

Ca^2+^ plays a central role in cell signaling at numerous levels. The tricarboxylic acid cycle consists of a series of reactions that produce energy through the breakdown of proteins, fatty acids and carbohydrates. Ca^2+^ within mitochondria regulates the most important task of the organelle: ATP production by oxidative phosphorylation. The physiological increase of mitochondrial Ca^2+^ stimulates the adenine nucleotide transporter (Mildaziene et al., 1995) and synthesis of ATP complex V (Das and Harris, 1990). Moreover, mitochondrial Ca^2+^ increase activates three matrix dehydrogenases: isocitrate dehydrogenase, α-ketogluterate dehydrogenase and pyruvate dehydrogenase (McCormack and Denton, 1979, 1993; McCormack et al., 1990). All three dehydrogenases enhance the reaction rate of many of the steps in the tricarboxylic acid cycle and therefore increase flux throughout the pathway, raising ATP production (Jouaville et al., 1999). Furthermore, it has been shown that motor neurons have an insufficient mitochondrial capacity to buffer large Ca^2+^ elevations which is partly due to a reduced mitochondrial density per volume compared to non-motor neurons (Grosskreutz et al., 2007). Mitochondrial disfunction and impaired Ca^2+^ homeostasis largely contribute to selective vulnerability of motor neurons (Jaiswal et al., 2009; Jaiswal and Keller, 2009).

#### Ca^2+^ overload and activation of permeability transition pore

One main mediator of mitochondrial function or dysfunction in neurons is the mPTP, which is a Ca^2+^ dependent high-conductance channel in the inner membrane of mitochondria (Brenner and Moulin, 2012). The mPTP comprises of the voltage-dependent anion channel (VDAC), the adenine nucleotide translocator (ANT) and cyclophilin D. Since VDAC and ANT are not essential for functioning of mPTP regulator (Juhaszova et al., 2008), the soluble matrix protein cyclophilin D received special attention (Giorgio et al., 2010). The mPTP opening is promoted by binding of cyclophilin D to the inner mitochondrial membrane (Di Lisa and Bernardi, 2009) and is favored by Ca^2+^ overload, ROS, inorganic phosphate and mitochondrial depolarization (Crompton, 1999; Brustovetsky et al., 2002; Bernardi et al., 2006). Binding of cyclophilin D to the inner mitochondrial membrane can be prevented by Cyclosporine A. Opening the mPTP causes a release of cytochrome C, which subsequently leads to apoptotic cell death (Liu et al., 1996; Crompton et al., 1998). High concentrations of cyclophylin D were found in swollen mitochondria in the SOD1 animal model. Modifying mPTP through different genetic and pharmacological manipulations has been shown to be protective in animal models of ALS. Genetic ablation of cyclophilin D delayed disease onset and extended the lifespan in the ALS mouse model (Martin et al., 2009). However, in another study, deleting cyclophilin D in the SOD1 mouse model did not lead to prolongation of survival, although it improved mitochondrial buffering capacity and attenuated mitochondrial damage (Parone et al., 2013). Therefore the role of cyclophylin D as a potential therapeutic is not fully understood.

#### Therapeutic strategies focused on mitochondrial Ca^2+^ dynamics

**FIGURE 4:**
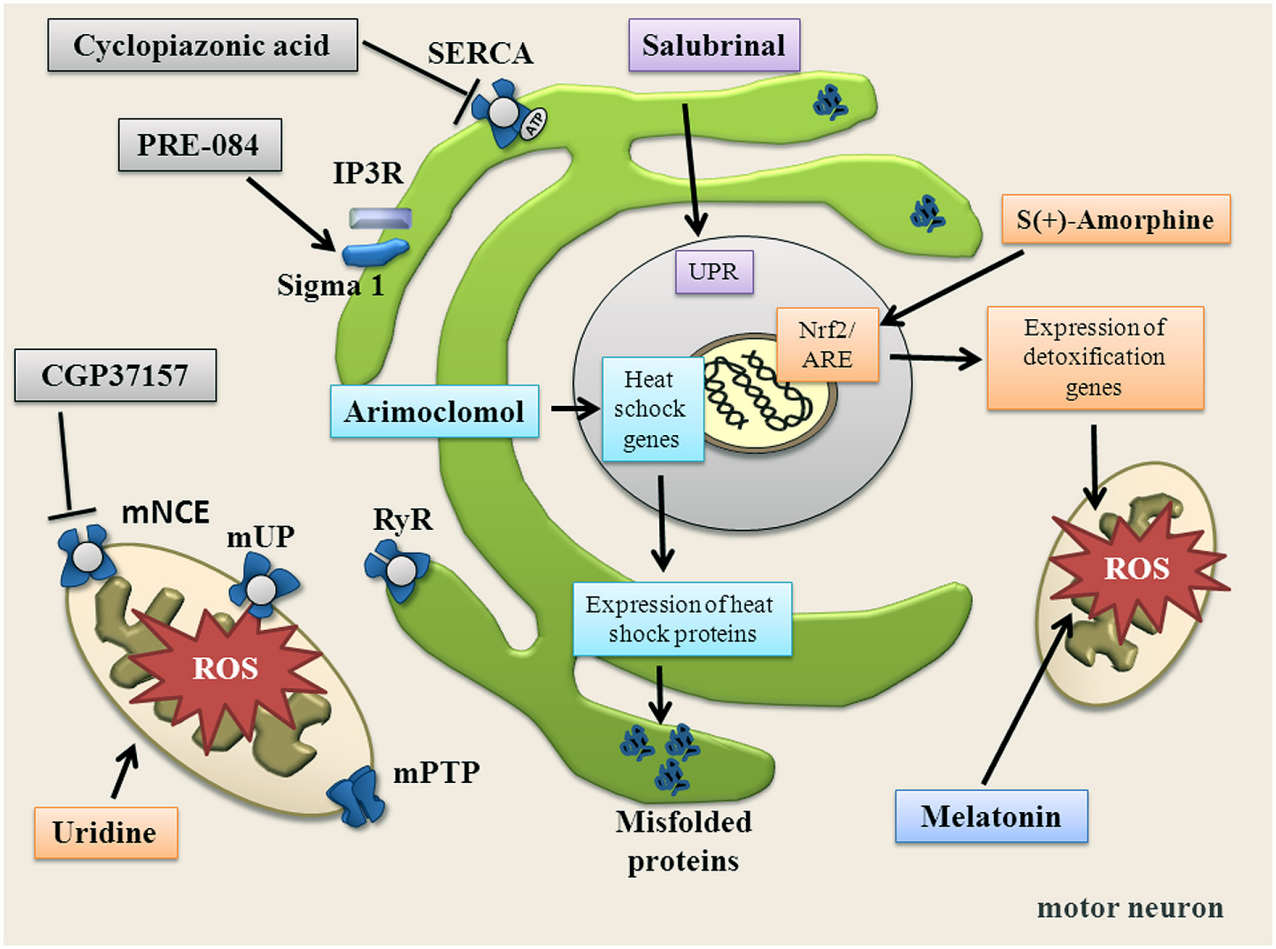
**Summary of substances targeting ER, mitochondria, oxidative stress and altered Ca^**2+**^ homeostasis.** Salubrinal increases the unfolded protein response (UPR) via phosphorylation of elF2α and activation of PERK. Arimoclomol induces heat shock response. PRE-084 activates Sigma-1 receptor. Uridine increases bioenergetic effects, increases ATP and enhances glycolytic energy production. CGP37157 blocks mNCE. Cyclopiazonic acid blocks SERCA. S(+)-Apomorphine activates the Nrf2/ARE pathway. Melatonin directly scavenges free radicals.

Because there is growing evidence for mitochondrial dysfunction in ALS, mitochondria are promising therapeutic targets (**Table 2**; **Figure 4**). However, studies targeting mitochondria have failed so far. The mPTP modulator Olesoxime (TRO19622) had a neuroprotective effect in motor neuron cell culture and in ALS rodents (Bordet et al., 2007; Martin, 2010a; Sunyach et al., 2012), but failed in a phase III clinical trial (http://www.als.net/ALS-Research/Olesoxime/ALS-Topics/). Further, Dexpramipexole, which reduces mPTP opening and increases cellular energy supply, did not have significant effects on survival and disease progression in a recent clinical trial (Cudkowicz et al., 2013).

**Table 2:** Substances targeting mitochondria.

Proposed mechanism	ALS model	Patients	Experimental evidence	Reference
**Mitochondrial targets**
Uridine	Improves bioenergetic effects, increases ATP levels and enhances glycolytic energy production	SOD1G93A mice	Increased survival, ameliorated body weight loss, enhanced motor performance, and decreased reactive astrogliosis	Amante et al. (2010)

CGP37157	Inhibitor of mNCE	SOD1G93A mice motor neurons	Protective effects against kainate induced excitotoxicity	Lautenschlager et al. (2013)
		SOD1G37R N2 cells	Restored Ca^2+^ levels upon application of bradykinin	Coussee et al. (2011)

Minocycline and creatine, compounds that improve mitochondrial function have also failed in human trials (Shefner et al., 2004; Gordon et al., 2007). Other medications targeting mitochondria such as coenzyme Q and cylosporin A have been studied, but all trials in humans were negative (Appel et al., 1988; Kaufmann et al., 2009). Uridine, a pyrimidine nucleoside, extended survival in SOD1G93A mice, probably by improving bioenergetic effects, increasing ATP levels, and enhancing glycolytic energy production (Amante et al., 2010). However, it has not been tested in ALS patients yet.

Melatonin recently gained interest because of its ability to decrease cytochrome C release and caspase-3 activation. It delayed disease onset in the SOD1G93A mice model. Besides its mitochondria stabilization effects in ALS, melatonin attenuated the activation of astrocytes and microglia (Zhang et al., 2013).

CGP37157, which is able to cross blood-brain barrier (Gonzalez-Lafuente et al., 2012), blocks the mNCE. It showed protective effects against kainate induced excitotoxicity in SOD1G93A mice motor neurons (Lautenschlager et al., 2013), restored calcium levels in SOD1G37R N2 cells (Coussee et al., 2011) and protected rat hippocampal slices upon veratridine-induced sodium and calcium overload (Gonzalez-Lafuente et al., 2012).

The transport of ADP, ATP and inorganic phosphates across mitochondrial membranes is regulated by the VDAC at the outer mitochondrial membrane. VDAC is regulated by Bcl-2 and both can form toxic complexes with mutated SOD1 (Pasinelli et al., 2004; Arbel and Shoshan-Barmatz, 2010; Israelson et al., 2010; Pedrini et al., 2010). Interactions between VDAC1, Bcl-2 and mutated SOD1 inhibits the conductance of VDAC1, leading to cell death (Israelson et al., 2010; Pedrini et al., 2010). It was demonstrated that small SOD1-like therapeutic peptides specifically block the formation in symptomatic SOD1G93A mice by restoring mitochondrial ADP permeability (Tan et al., 2013).

Human TDP-43 caused mitochondrial morphologic abnormality and decrease of mitochondrial complex I activity, and mitochondrial transmembrane potential in human TDP-43 stably tranfected NSC-34 cells. Dimethoxy curcumin was able to ameliorate mitochondrial dysfunction in the same experiment, which makes this drug interesting as a potential therapeutic for TDP-43 linked ALS (Lu et al., 2012).

### CALCIUM AND MITOCHONDRIAL ROS

Mitochondria are the main sites of ROS formation as by-products of ATP production (Coyle and Puttfarcken, 1993; Brand, 2010). However, mitochondrial Ca^2+^ overload and abnormal oxidative phosphorylation increase ROS production and oxidative stress (Carriedo et al., 2000; Mattiazzi et al., 2002; Murphy, 2009; **Figure 3**). Many of the stated mitochondrial respiratory abnormalities have been linked to reduced activity of mitochondrial complexes I and IV (Mattiazzi et al., 2002; Rizzardini et al., 2006; Son et al., 2008; Coussee et al., 2011).

#### Reactive oxygen species and oxidative stress

Moderate levels of ROS and reactive nitrogen species (RNS) promote cellular proliferation, regulation and survival. Typical ROS are free radical species such as superoxide ($O_{2}^{•-}$), hydroxyl radicals (^•^OH) and non-radical species like hydrogen peroxide (H_2_O_2_). The respiratory chain complexes I and III are the primary mitochondrial sources of $O_{2}^{•-}$ Oxidative stress has been implicated as a pathological mechanism of both fALS and sALS (Ferrante et al., 1997). ROS has been detected in the spinal cord and cerebrospinal fluid of sALS patients (Tohgi et al., 1999). Increased H_2_O_2_ and oxidative damage to protein and DNA were observed in mutated SOD1 transgenic mice (Liu et al., 1999). Many ALS causing genes and genes modifiers are known to influence ROS production (Carter et al., 2009). SOD1 mutation induces oxidative modifications of several proteins in ALS: SOD1, translationally controlled tumor protein (TCTP), ubiquitin carboxyl-terminal hydrolase-L1 (UCH-L1) and probably alphaB-crystallin. These oxidative modifications lead to structural alteration and a decline of protein activity (Poon et al., 2005). ROS also directly influences transcriptional factors such as NF-kB, activator protein 1 (AP-1), and HIF-1α which are all involved in the regulation of gene expression and maintaining cellular homeostasis (Haddad, 2002).

ROS dysregulation of these factors is observed in ALS pathology (Iaccarino et al., 2011; Moreau et al., 2011) as the involvement of protein disulfide isomerase (PDI) family members plays an important role in oxidative folding of human secretory proteins (Rutkevich et al., 2010; **Figure 2**). PDI’s enzymatic activity can be inactivated by oxidation and *S*-nitrosylation of their active site thiol groups. In motor neurons of patients with ALS, PDI was widely distributed and aggregated (Atkin et al., 2008). Therefore it was assumed that PDI is inactivated due to S-nitrosylation in the affected neurons, which causes protein misfolding in ALS (Honjo et al., 2011). Studies of genetics, model organisms, and patient’s tissue samples support PDI upregulation triggered by ER stress and post-translational inhibition of PDI due to *S*-nitrosylation (Atkin et al., 2008; Walker and Atkin, 2011).

#### Therapeutic strategies focused on reactive oxygen species

Design of novel antioxidant strategies to selectively target oxidative stress and redox imbalance might be an important approach (**Table 3**; **Figure 4**). However, the antioxidant treatment therapy in ALS has not been successful so far (Traynor et al., 2006; Bedlack et al., 2007; Beghi et al., 2011). SOD1 is tightly connected with the nuclear erythroid 2-related-factor 2 (Nrf2). Nrf2 is a transcriptional factor and main expression regulator of many antioxidant/detoxification genes via its interaction with the antioxidant response element (ARE). Because it helps neuronal cells to cope with toxic effect of oxidative stress, pharmacological targeting of Nrf2/ARE pathway was proposed as a tool against neurodegeneration in ALS (Petri et al., 2012; Milani et al., 2013; **Figure 4**). S(+9)-Apomorphine, a CNS penetrating activator of the Nrf2/ARE pathway was able to reduce pathological oxidative stress and improved survival in fibroblasts of ALS patients, and also slowed disease progression in SOD1G93mice (Mead et al., 2013). On the other hand, Guo et al. (2013) reported a rather modest impact of Nrf2 on the course of disease in SOD1G93A mice.

**Table 3:** Substances targeting ROS.

Proposed mechanism	ALS model	Patients	Experimental evidence	Reference
**ROS targets**
S (+)-Apopomorphine	Activator of the Nrf2/ARE pathway	Fibroblasts from ALS patients		Protection against menadione-induced cell death and reduction in basal oxidative stress was observed in fibroblasts from ALS patients when treated with *S*[+]-apomorphine	Mead et al. (2013)
		SOD1G93A mice		S(+)-apomorphine demonstrated CNS penetrance, Nrf2 induction and significant attenuation of motor dysfunction	
Melatonin	Acts against oxidative and nitrosative stress-induced damage	NSC-34		Attenuated glutamate-induced cell death of NSC-34	Weishaupt et al. (2006)
		SOD1G93A mice		Delayed disease progression and extended survival of SOD1 mice	
			ALS patients	Decreased oxidative stress in patients, High-dose (300~mg/day) rectally administered melatonin was well-tolerated in patients with sporadic ALS	
		SOD1G93A mice		Delayed disease onset, neurological deterioration and mortality	Zhang et al. (2013)
			ALS patients	Daily oral melatonin administration in ALS patients was well tolerated	Jacob et al. (2002)

Beneficial effects have been reported in NSC-34 cells for melatonin, a hormone which acts against oxidative and nitrosative stress-induced damage (Weishaupt et al., 2006). In several studies, treatment with melatonin prolonged survival in the SOD1G93A mice (Weishaupt et al., 2006; Zhang et al., 2013), however, Dardiotis et al. (2013) showed contrary results in the same mouse model, possibly because melatonin exacerbated neurodegeneration.

In patients with ALS, high doses of melatonin were well tolerated (Jacob et al., 2002; Weishaupt et al., 2006) and it was reported that circulating serum protein carbonyls, which are oxidative stress markers, were decreased in melatonin treated ALS patients (Weishaupt et al., 2006).

Diacetylbis(*N*(4)-methylthiosemicarbazonato) copper(II), that inhibits the action of peroxynitrite on SOD1 and ensues nitration of cellular proteins, significantly delays onset of paralysis, prolongs lifespan and prevents accumulation of TDP-43 in the spinal cord of SOD1G93A mice. Therefore, it represents a potential neuroprotective agent targeting multiple disease pathways in ALS (Soon et al., 2011). In the spinal cord of ALS patients, metallothioneins (Zn modulators and anti-oxidant reaction inducers), were seriously reduced (Hozumi et al., 2008). It was demonstrated that metallothionein-III prevents the loss of motor neurons and prolongs the life span of ALS mice (Hashimoto et al., 2011; Hozumi, 2013).

### CALCIUM HOMEOSTASIS AND ROS

Ca^2+^ plays a role in ROS production and, vice versa, the redox state can modulate Ca^2+^ signaling. Components of ROS homeostasis are regulated by Ca^2+^-dependent pathways. Ca^2+^ stimulates NO synthesis, inhibits complex IV, and leads to ROS production at the complex III (Feissner et al., 2009). Depending on the targeted protein, the type and concentration of ROS and the duration of exposure, interactions between Ca^2+^ and ROS signaling can be stimulating or inhibiting.

#### Ryanodine receptors are stimulated by oxidation

Ryanodine receptors (RyR) belong to a class of intracellular Ca^2+^ channels and are important mediators of Ca^2+^ induced Ca^2+^ release in excitable cells such as muscles and neurons (Fabiato, 1983; McPherson et al., 1991). RyR are opened by Ca^2+^ itself which may induce propagated Ca^2+^release on the ER surface. Ca^2+^ entry through AMPA receptors caused RyR mediated Ca^2+^-induced Ca^2+^ release from the ER in embryonic motor neurons in co-culture with neonatal Schwan cell (Jahn et al., 2006). In embryonic motor neurons, Ca^2+^-induced Ca^2+^ release was shown to contribute greatly to AMPA receptor stimulation induced Ca^2+^-induced Ca^2+^ release through RyR and Ca^2+^ dysregulation (Grosskreutz et al., 2004). RyR form tetramers in the sarcoplasmic reticulum (SR) and ER membranes (Xu et al., 1998; Fill and Copello, 2002). The reversible oxidation of endogenous SH groups opens the channel and releases Ca^2+^ from SR (Abramson and Salama, 1989; Xu et al., 1998). Since sulfhydryl oxidation of reactive thiols is involved in the gating of the Ca^2+^ release channel, RyR represents an important target in oxidative cell damage (Liu et al., 1994; Zable et al., 1997; Marengo et al., 1998). Activity of the RyR channel complex is regulated as a response to changes in transmembrane redox potential (Feng et al., 2000). When Ca^2+^ channel activators lower the redox potential of the RyR, the thiol groups get oxidized and the channel opens. When Ca^2+^ channel inhibitors increase the redox potential of the RyR, the disulfides are reduced and the channel closes (Feng et al., 2000). ROS, such as $O_{2}^{•-}$ and H_2_O_2,_ can activate the channel by direct oxidation of redox-sensing thiols (Rowe et al., 1983; Boraso and Williams, 1994; Zima et al., 2004). The endogenous ligand of RyR, FKBP12, stabilizes RyR in the absence of activation and prevents Ca^2+^ leakage from the ER. The concentration of FKBP12 was decreased in ALS patients indicating the importance of equilibrium between FKBP12 and RyR in neurodegeneration (Kihira et al., 2005).

#### IP_3_R are stimulated by oxidation

A second receptor that induces the release of Ca^2+^ from the ER is the IP_3_R. Ca^2+^ overload in the ER discharges IP_3_R spontaneously (Missiaen et al., 1991; Rooney et al., 1991). The most important ligands that modulate IP_3_R channel activity are InsP3 and Ca^2+^. At low concentrations Ca^2+^ activates the channel, whereas at higher concentrations, Ca^2+^ inhibits the channel (Foskett et al., 2007). IP_3_R can be directly activated by oxidative agents, such as $O_{2}^{•-}$ (Suzuki and Ford, 1992) and H_2_O_2_ (Wesson and Elliott, 1995). Thimerosal, a sulfhydryl-oxidizing agent, stimulates IP_3_R channels isolated from rat cerebellum and incorporated into artificial lipid bilayer (Thrower et al., 1996) and HeLa cells (Bootman et al., 1992). Overexpression of the IP_3_R2 shortened the lifespan in SOD1G93A mice, which implicates the importance of ER Ca^2+^ release by IP_3_Rs and that impaired function of this receptor can be destructive in ALS (Staats et al., 2012a). IP_3_-gated Ca^2+^ seems to be a key regulator of TDP-43 nucleoplasmic shuttling and proteostasis. Pathologic TDP-43 aggregation disturbs Ca^2+^-dependent TDP-43 shuttling, indicating pharmacological manipulation of IP_3_R as a target in TDP-43 induced neurodegeneration *in vivo* (Kim et al., 2012).

Phospholipase C delta 1 (PLCδ1) increases InsP_3_ formation which releases calcium from ER through IP_3_R. The expression of PLCδ1 is increased in ALS mouse spinal cord and neurons. Genetic ablation of PLCδ1 prevented shrinkage of motor neurons in ALS mice, suggesting that PLCδ1 is also a candidate for new targets in ALS research (Staats et al., 2013).

#### SERCA and plasma membrane Ca^2+^-ATPase are inhibited by oxidation

SERCA transfers Ca^2+^ from the cell cytosol to the lumen of the SR (MacLennan et al., 1997). SERCA is very sensitive to redox state but contrary to RyR and IP_3_R, oxidation inhibits SERCA activity (Kaplan et al., 2003). SERCA is reversibly regulated through NO-dependent S-glutathiolation of specific cysteine residues (Adachi et al., 2004), where irreversible sulfonylation reduces SERCA (Ying et al., 2008). Thiol oxidizing agents inhibit and glutathione stimulate SERCA (Scherer and Deamer, 1986). Amino acid peroxides selectively oxidize cysteine residues of SERCA and inactivate the pump (Dremina et al., 2007). $O_{2}^{•-}$ and H_2_O_2_/^•^OH have been shown to inhibit Ca^2+^ uptake into the sarcoplasmic reticulum (Rowe et al., 1983; Kukreja et al., 1988; Xu et al., 1997). H_2_O_2_/^•^OH directly interfere with the ATP binding site. Since the Ca^2+^ uptake into ER is coupled to the ATP hydrolysis, restriction of ATPase activity decreases Ca^2+^ uptake (Xu et al., 1997). Ca^2+^ transport and ATPase activity of plasmalemmal Ca^2+^ ATPase can be inhibited by ROS due to oxidation of SH groups and peroxidation of membrane phospholipids (Kaneko et al., 1989). In SOD1G93A motor neurons, ER Ca^2+^ uptake by SERCA was shown to be increased (Lautenschlager et al., 2013). Ca^2+^ handling is reshaped during disease progression in the SOD1G93A mouse model. Increased plasma membrane extrusion upon mitochondrial failure likely indicates a compensatory mechanism in the disease. This study puts the focus on further investigations of mitochondrial and plasma membrane Ca^2+^ transporters such as plasmalemmal Ca^2+^ ATPase and plasma membrane Na^+^/Ca^2+^ exchanger (Fuchs et al., 2013).

#### Therapeutic strategies targeting RyR, IP_3_R, and SERCA

Blocking RyR using dantrolene has provided protection of motor neurons exposed to a brief excitotoxic insult *in vitro*, but did not show a protective effect in SOD1G93A mice. This indicates that Ca^2+^ release through RyRs have a modest role in SOD1 mice (Staats et al., 2012b). Inhibiting SERCA by cyclopiazonic acid showed protective effects against kainate induced excitotoxicity in SOD1G93A cultured motor neurons (Lautenschlager et al., 2013). Although there are not many studies targeting ERMCC Ca^2+^ channels, these could be valuable targets for further investigation.

## CONCLUDING REMARKS

Riluzole is currently the only approved drug for ALS, but at best it only slows disease progression for some months. It is crucial to understand disease pathophysiology and to recognize the major upstream events that lead to motor neuron death. Disturbances of Ca^2+^ homeostasis and ER function are well-known features of motor neuron degeneration in ALS. Dysregulation in between the ERMCC is therefore characterized accumulation of misfolded proteins, oxidative stress and motor neuron death. Therapeutic drugs aiming to stabilize the ERMCC, reduce ER stress and support the UPR may be effective in a wide range of neuron diseases. However, genetic and the majority of pharmacologic strategies to protect ER and mitochondria against exitotoxicity have been unsuccessful. Nevertheless, these negative results, added to the many failed trials in the past, raise the question of the suitability of our experimental models, which are mostly murine. Perhaps we should focus on new tools such as induced pluripotent stem cells taken from ALS patients and derived into motor neurons. They could generate more suitible models.

Newly discovered genes that cause ALS may also offer new therapeutics for ALS. These strategies are currently underway. The SOD1G93A mice model has been extensively investigated so far, but there is an urgent requirement for additional models of ALS such as TDP-43, FUS and VCP. Moreover, the drugs that failed in clinical trials could still prove to play a valuble role as part of a combination strategy with other molecules in the future, such as drugs that operate in distinct or overlapping pathways. Developing of “smart drugs”, such as Arimoclomol that enhance protein folding capacity only under conditions of cellular stress, may also be good direction in drug development.

Finally a significant point comprises establishing improved pharmacokinetic profiles. The safety properties and most efficient dose of the drug in humans have to be adequately established prior to phase III trials. Taken together, the key for success is in basic and clinical researchers continuing to work together.

## AUTHOR CONTRIBUTIONS

All authors contributed in the conception and design of the present review, as well as in drafting and revising the manuscript.

## Conflict of Interest Statement

The authors declare that the research was conducted in the absence of any commercial or financial relationships that could be construed as a potential conflict of interest.

## ABBREVIATIONS

ALS: amyotrophic lateral sclerosis
AMPA: α-amino-5-methyl-3-hydroxisoxazolone-4-propionate
ANT: adenine nucleotide translocator
AP-1: activator protein 1
ARE: antioxidant response element
ATF6: basic leucine-zipper transcription factor 6
Bax/Bak: Bcl-2-associated X protein
Bcl-2: B-cell lymphoma 2 protein
BIK: Bcl-2 interacting killer protein
*C9ORF72*: chromosome 9 open reading frame 72
CHOP: transcription factor C/EBP homologous protein
eIF2α: eukaryotic initiation factor-2
ER: endoplasmic reticulum
ERMCC: endoplasmic reticulum mitochondria calcium cycle
fALS: familial amyotrophic lateral sclerosis
FUS/TSL: fused in sarcoma/translated in liposarcoma
HIF-1α: hypoxia-induced factor
Hsf1: heat shock transcription factor 1
InsP3: inositol 1,4,5-trisphosphate
IP_3_R: inositol 1,4,5-triphosphate receptor-gated channel
IRE1: inositol-requiring enzyme 1
mNCE: mitochondrial sodium calcium exchanger
mPTP: mitochondrial permeability transition pore
mUP: mitochondrial uniporter
NF-κB: nuclear factor kappa-light-chain-enhancer of activated B cells
NO: nitric oxide
Nrf2: erythroid 2-related-factor 2
*OPTN*: optineurin
PDI: protein disulfide isomerase
PERK: the double-stranded RNA-activated protein kinase (PKR)-like ER kinase
PLCδ1: phospolipase C delta 1
ROS: reactive oxygen species
RyR: ryanodine receptors
sALS: sporadic amyotrophic lateral sclerosis
SERCA: sarco/endoplasmic reticulum Ca^2+^ ATPase
SOD1: Cu/Zn superoxide dismutase type 1
SR: sarcoplasmic reticulum
*TARDBP*: TAR DNA binding protein
TCTP: translationally controlled tumor protein
TDP-43: transactive response DNA binding protein 43 kDa
UBQLN2: ubiquilin-2
UCH-L1: ubiquitin carboxy-terminal hydrolase L1
UPR: unfolded protein response
*VAPB*: vesicle-associated membrane protein (VAMP)-associated protein B
*VCP*: valosin-containing protein
VDAC: voltage-dependent anion channel.
